# Integrative analysis of different low-light-tolerant watermelon lines in response to low-light stress

**DOI:** 10.1186/s12870-025-07180-8

**Published:** 2025-08-21

**Authors:** Xiaojing Ma, Yong Wang, Xiaohui Li, Yongpeng Liu, Hengbin Luo, Wenkai Shang, Yancui Di, Ningning Gao, Liyun Kang, Fengzhi Piao, Zhixin Guo, Han Dong, Weixing Zhao, Tao Zhang

**Affiliations:** 1 College of Horticulture, Henan Agricultural University, Zhengzhou, 450002 P. R. China; 2 Research Institute of Henan Academy of Agricultural Sciences, Zhengzhou, 450002 P. R. China; 3 Luohe Academy of Agricultural Sciences, Luohe, 462000 P. R. China

**Keywords:** Antioxidant enzymes, Auxin expression, Gene expression, Integrative analysis, Low-light stress, Light-harvesting complex

## Abstract

Low-light stress is a major environmental factor limiting watermelon growth and productivity; however, the molecular and physiological mechanisms underlying low-light tolerance remain unclear. In this study, we compared the morphological, physiological, biochemical, and transcriptomic responses of two watermelon inbred lines, HY25 (low-light tolerant) and DQ22 (low-light sensitive), under low-light conditions (60 µmol m⁻² s⁻¹) and normal light (200 µmol m⁻² s⁻¹). Morphological assessments revealed that low-light stress considerably inhibited shoot and root development in both lines, with DQ22 exhibiting more severe reductions in plant height, biomass, and root architecture. Photosynthetic analysis revealed that, compared with HY25, DQ22 resulted in greater decreases in the net photosynthetic rate, stomatal conductance, transpiration rate, chlorophyll content, and PSII efficiency (Fv/Fm). Biochemical assays showed that DQ22 resulted in increased levels of reactive oxygen species (ROS), malondialdehyde (MDA), and relative electrolyte leakage (REL), whereas HY25 maintained increased antioxidant enzyme activity and decreased cellular damage. Additionally, HY25 resulted in greater retention of carbohydrate and protein contents under stress. Transcriptome analysis revealed 8,291 differentially expressed genes (DEGs), with HY25 exhibiting increased expression of genes related to photosynthesis, carbohydrate metabolism, and ROS detoxification. Notably, genes involved in light-harvesting complexes, carbon fixation, and porphyrin metabolism were more actively expressed in HY25. Moreover, auxin-related genes presented genotype-specific expression under stress. These findings clarify key physiological traits and gene regulatory mechanisms associated with low-light tolerance in watermelon and provide valuable insights for developing stress-resistant cultivars.

**Supplementary Information:**

The online version contains supplementary material available at 10.1186/s12870-025-07180-8.

## Introduction

Watermelon (*Citrullus lanatus*), a highly nutritious fruit comprising 93% water with small quantities of protein, fat, minerals, and vitamins, is one of the most popular fruits in the world and is widely produced in tropical and subtropical regions, accounting for 7% of the world’s vegetable production, second only to tomatoes [1–3]. China has led the world in terms of both watermelon cultivation area and yield for decades, producing 60.86 million tons in 2021—approximately 60% of the global output [4, 5]. Watermelon is a photophilic crop, and its growth and development are substantially hindered under low-light conditions [6, 7]. Owing to global climate change, the frequency of extreme weather events—particularly prolonged cloudiness and rainfall—has increased, resulting in sustained low-light environments in protected cultivation systems [8]. In northern China and similar regions, low-light stress has become a major limiting factor for greenhouse watermelon production [9, 10].

Light is one of the most critical environmental signals and not only provides energy for photosynthesis but also regulates various growth processes throughout the life cycle of plants [11, 12]. In greenhouse cultivation, low-light intensity is one of the main factors limiting the growth, development, and yield of crops [13–15]. Low-light stress affects the root activity of watermelon, inhibits growth and development, causes seedlings to grow weak or even die, and severely harms watermelon cultivation [6, 16]. Low-light stress also disrupts physiological processes, leading to decreased photosynthetic efficiency, reduced carbon fixation, ROS overaccumulation, and membrane lipid peroxidation [15, 17–19].

Low-light stress leads to the formation of weak cucumber seedlings with small leaves, long stems, and fewer female flowers [9, 20]. Continuous low-light stress can decrease photosynthesis in leaves by disrupting photosynthetic organelles [21, 22]. In tomato, reduced light transmittance decreases photosynthesis and increases O₂⁻ and H₂O₂ levels, accelerating photoinhibition of PSII [23, 24]. Low-light stress disrupts the homeostasis of hormones; decreases the contents of indole acetic acid (IAA), zeatin riboside (ZR), and gibberellin (GA) in maize seedlings; increases the content of abscisic acid (ABA); and inhibits the growth and development of grains [25]. Similarly, in corn, low light lowers starch and amylose contents, increases protein accumulation, and negatively affects grain quality [26].

Plants counteract low-light stress through a combination of physiological, biochemical, and molecular mechanisms [20]. For example, chloroplast morphology and pigment content in cucumber change considerably under low-light conditions [27, 28], and in soybean, sensitive varieties show increased cell elongation, whereas tolerant varieties maintain better photosynthetic efficiency and yield under stress [29]. Therefore, investigating the mechanisms of low-light tolerance is essential for breeding watermelon cultivars adapted to such conditions and improving protected cultivation outcomes.

In watermelon (*Citrullus lanatus*), understanding the mechanisms underlying resistance to low-light stress is essential, particularly with respect to photosynthetic performance, chlorophyll metabolism, and the expression of photosynthesis-related genes. However, comprehensive comparative analyses of the photosynthetic capacity of low-light-tolerant and low-light-sensitive lines remain limited. In particular, the physiological and molecular strategies by which tolerant genotypes mitigate the adverse effects of low-light stress are poorly understood. In this study, we evaluated the photosynthetic phenotype, conducted transcriptome (RNA-seq) analysis, and assessed the expression of photosynthesis-related genes in the leaves of contrasting watermelon lines under low-light conditions. Our findings reveal how low-light stress influences photosynthetic efficiency, the accumulation of photosynthetic products, and hormone-associated responses. This research provides important insights into the physiological and molecular basis of low-light tolerance in watermelon and offers a theoretical foundation for the breeding and improvement of light-resistant cultivars.

## Materials and methods

### Plant materials and growth conditions

Seeds of two homozygous watermelon inbred lines DQ22 and HY25 were provided by Prof. Weixing Zhao at Horticultural Research Institute of Henan Academy of Agricultural Sciences. Both lines were previously screened by Prof. Zhao’s team for low-light tolerance. HY25 is a low-light-tolerant line that grows well under low-light stress, whereas DQ22 is a low-light-sensitive line that exhibits abnormal growth, including chlorosis and terminal flowering.

The experiments were carried out at the Facility Engineering and Structure Laboratory of Henan Agricultural University (113.79°N, 34.79°E). Watermelon plants were planted in pots (height of 8 cm, diameter of 9 cm, and each pot contained one watermelon seedling) filled with 250 g of vermiculite (1–3 mm, pH 7.5), and were supplied with Hoagland’s nutrient solution (0.36 g/L Ca(NO_3_)_2_, 0.10 g/L KH_2_PO_4_, 0.80 g/L KNO_3_, 0.04 g/L NH_4_NO_3_, 0.13 g/L MgSO_4_, and 0.01 mg/L of Mikron fertilizer) every 3 days. The growth conditions were kept as follows: a day/night temperature of 25/20℃ and a 12-h photoperiod (6AM–6PM).

### Light treatment

The photosynthetic photon flux density (PPFD) was maintained at 200 µmol m⁻² s⁻¹ under white light. Illumination was provided by LED lamps (Henan Zhishengpu Electronic Technology Co., Ltd., China) with a red (R) to blue (B) light ratio of 3:2, unless otherwise specified. Low-light treatment (low light, LL) was applied at the two-leaf stage, with a light intensity of 60 µmol m⁻² s⁻¹, whereas normal conditions (control, CK) were maintained at 200 µmol m⁻² s⁻¹.

### Determination of morphological parameters

After low-light stress treatment for 25 d, the growth indicators of watermelon seedlings were assayed. The shoot morphological parameters were measured as follows: plant height was measured from the base of the stem to the tip of the longest leaf using a ruler; stem diameter was measured at the midpoint using a digital calliper; leaf length was recorded from the base to the tip of the largest fully expanded leaf; petiole length was measured from the stem attachment point to the leaf blade base; whole plant fresh weight was obtained after uprooting, washing, and immediate weighing on an electronic balance; and dry weight was measured after oven drying at 70 °C for 48 h. For root analysis, the roots were gently washed and photographed for phenotype assessment, while the total root length, surface area, and average diameter were measured using a plant root analysis system (WinRhizo, REGENT Instruments, Inc., Canada), and the root volume was determined via the displacement method or using root analysis software. To analyse the length of the main root, we first washed the root with clean water, then laid it flat on a table and measured the length directly from the root tip to the root tip with a ruler. All the measurements were performed with four biological replicates per treatment.

### Determination of chlorophyll fluorescence, photosynthetic capacity, and chlorophyll content in watermelon lines under low-light tolerance

The maximum potential PSII efficiency (Fv/Fm) was measured using a FluorCam7 Chl fluorescence imaging (Photon Systems Instruments, USA) according to previously described methods [30]. Leaves were dark-adapted for 30 min prior to measurement using leaf clips. After dark adaptation, the minimal fluorescence (Fo) was measured using weakly modulated light. A saturating pulse of light (3000 µmol m⁻² s⁻¹ for 0.8 s) was then applied to determine the maximal fluorescence (Fm). The variable fluorescence (Fv) was calculated as Fm – Fo, and the maximum quantum yield was determined using the formula: Fv/Fm= (Fm − Fo)/Fm.

The photosynthesis parameters were measured the 3rd fully expanded leaves after treatment with low light for 25 d at 9:00–10:00 AM. The net photosynthetic rate (Pn), stomatal conductance (Gs), intercellular CO_2_ concentration (Ci) and transpiration rate (Tr) were determined using an LI-6400 photosynthetic apparatus (LICOR Inc., NE, USA) as previously described [31]. Furthermore, the *Chl* contents in watermelon seedling leaves were detected by the ethanol–acetone method [32].

### Determination of MDA content, relative electrolyte leakage (REL) and antioxidant enzyme activity

As previously mentioned, the lipid peroxidation level of leaves was evaluated by determining the content of malondialdehyde (MDA) using the thiobarbituric acid (TBA) test [33].

Leaf membrane integrity was evaluated by measuring relative electrolyte leakage using a previously described method [34]. Approximately 0.2 g of fresh leaf discs (5–10 mm diameter) were excised and rinsed three times with deionized water to remove surface-adhered electrolytes. The samples were then incubated in 20 mL of deionized water in sealed test tubes and shaken gently at 25 °C for 2–3 h. After incubation, the initial electrical conductivity of the solution (C₁) was measured using a conductivity meter (DDS-307 A, Shanghai INESA Scientific Instrument Co., Ltd., China). The samples were subsequently subjected to boiling in a water bath at 99 °C for 30 min to ensure the complete release of all the electrolytes and cooled to room temperature, after which the final conductivity (C₂) was measured. The REL was calculated using the following formula: REL (%)=(C₁/ C₂) × 100%.

Twenty-five days after the low-light treatment, 0.2 g of leaves was harvested to detect enzyme activity. The activities of superoxide dismutase (SOD) and catalase (CAT) were detected as previously described [35, 36]. A Micro Peroxidase (POD) Assay Kit (BC0095, Beijing, China) was used to assay the activity of microperoxidase (POD).

### Determination of carbohydrate, soluble protein, and proline contents

The leaf tissue (0.2 g) of the tomato seedlings after low-light treatment for 25 d was collected for detection. The starch content was determined using a starch content detection kit (BC0705, Solarbio, Beijing, China). In accordance with previous methods [37], the soluble sugar content in the leaves was measured using the anthrone–sulfuric acid method. The soluble protein content in the leaves was assayed by an ultraviolet spectrophotometer at a wavelength of 595 nm after the extraction mixture was stained with Coomassie brilliant blue C-250 [38]. The proline content was measured with a proline (PRO) test kit (A107-1-1, Nanjing, China).

### RNA isolation, cDNA synthesis and transcriptome analysis

In this study, samples were taken from the 2nd fully expanded leaves after 48 h of low light treatment. According to a previously described [39], an RNA extraction kit (Quick RNA Isolation Kit, 0416–50, Hua Yue Yang, China) and the ReverTra Ace qPCR RT Kit (R223, Vazyme, China) were respectively used to extract the total RNA from watermelon leaves and reverse transcribe total RNA, respectively. The results of the qRT‒PCR analyses were detected on a Light Cycler^®^ 480 II Real-Time PCR Detection System (Roche, Basel, Switzerland) using the SYBR Green PCR Master Mix Kit (Q412, Vazyme, China). The relative expression levels were normalized to the expression level of the watermelon housekeeping gene *β-actin* (*ClACT*) The gene-specific primers used are listed in Supplemental Table S1.

The RNA samples that met the quality control requirements were sent to the Shanghai Paisenuo Biotechnology Co., Ltd for RNA-seq analysis via an Illumina HiSeq 2000 (USA/Illumina). The raw data (raw reads) were filtered with the FASTQ_Quality_Filter tool from the FASTX-toolkit, and used for further analysis. After preprocessing the RNA-seq data, the filtered reads were subsequently mapped to the reference sequences using SOAP2 aligner/soap2 [40] and subsequently mapped to the watermelon genome database (http://cucurbitgenomics.org/v2/ftp/genome/watermelon/97103/v2.5). Differentially expressed genes were identified based on the basis of an adjusted *p* value < 0.05 and a fold change > 1.5.

### Statistical analyses

Statistical analyses were performed using Statistics Package for Social Science (SPSS) version. 19.0. Differences between treatments were evaluated via Tukey’s test, with significance determined at *P* < 0.05, as indicated in the figure legends.

## Results

### Morphological observations of watermelon lines with different low-light tolerances

**Fig. 1:**
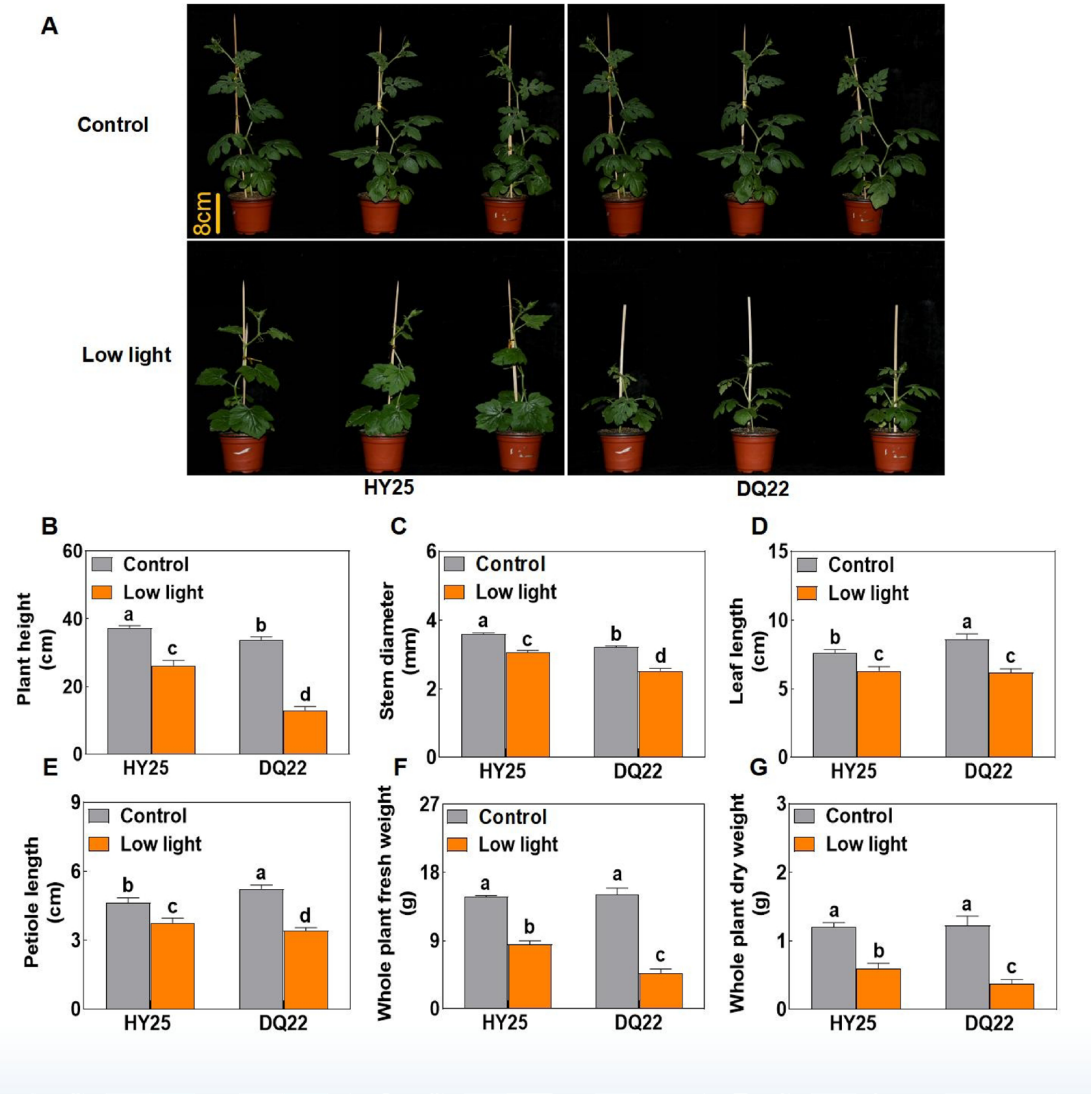
Morphological traits of watermelon seedlings under normal and low-light conditions. The plant performance (**A**), height (**B**), stem diameter (**C**), leaf length (**D**), petiole length (**E**), whole-plant fresh weight (**F**), and whole-plant dry weight (**G**) under normal light (control) and low-light stress conditions at 25 d in different watermelon lines. HY25: tolerant line. DQ22: sensitive line. The values are the means ± standard deviations (*n* = 4). Different letters indicate significant differences (*P* < 0.05) according to Tukey’s test

Under normal light conditions, both low-light-tolerant (HY25) and low-light-sensitive (DQ22) watermelon seedlings exhibited healthy growth with green leaves (Fig. 1A). Compared with those of HY25, the plant height and stem diameter of DQ22 seedlings were significantly lower, whereas the leaf length and petiole length were noticeably greater (Fig. 1B–E). However, no significant differences in whole-plant fresh weight or dry weight were detected between the two lines under normal light conditions (Fig. 1F, G). In contrast, significant differences in growth response were observed between the two lines under low-light stress. Morphological analysis revealed that although the growth of HY25 seedlings was inhibited by low light, the reduction was moderate. Specifically, plant height, stem diameter, leaf length, petiole length, fresh weight, and dry weight in HY25 were 30.16, 14.27, 16.84, 18.97, 42.41, and 50.41% lower (Fig. 1B–G), respectively, than those in plants grown under normal light. However, DQ22 plants exhibited a more severe response to low-light stress, including a dwarf phenotype, thinner stems, smaller leaves, and visible chlorosis. Compared with those under the control conditions, the plant height, stem diameter, leaf length, petiole length, fresh weight, and dry weight of DQ22 under low light conditions were reduced by 61.75, 21.53, 28.31, 34.48, 68.65, and 69.91%, respectively.

**Fig. 2:**
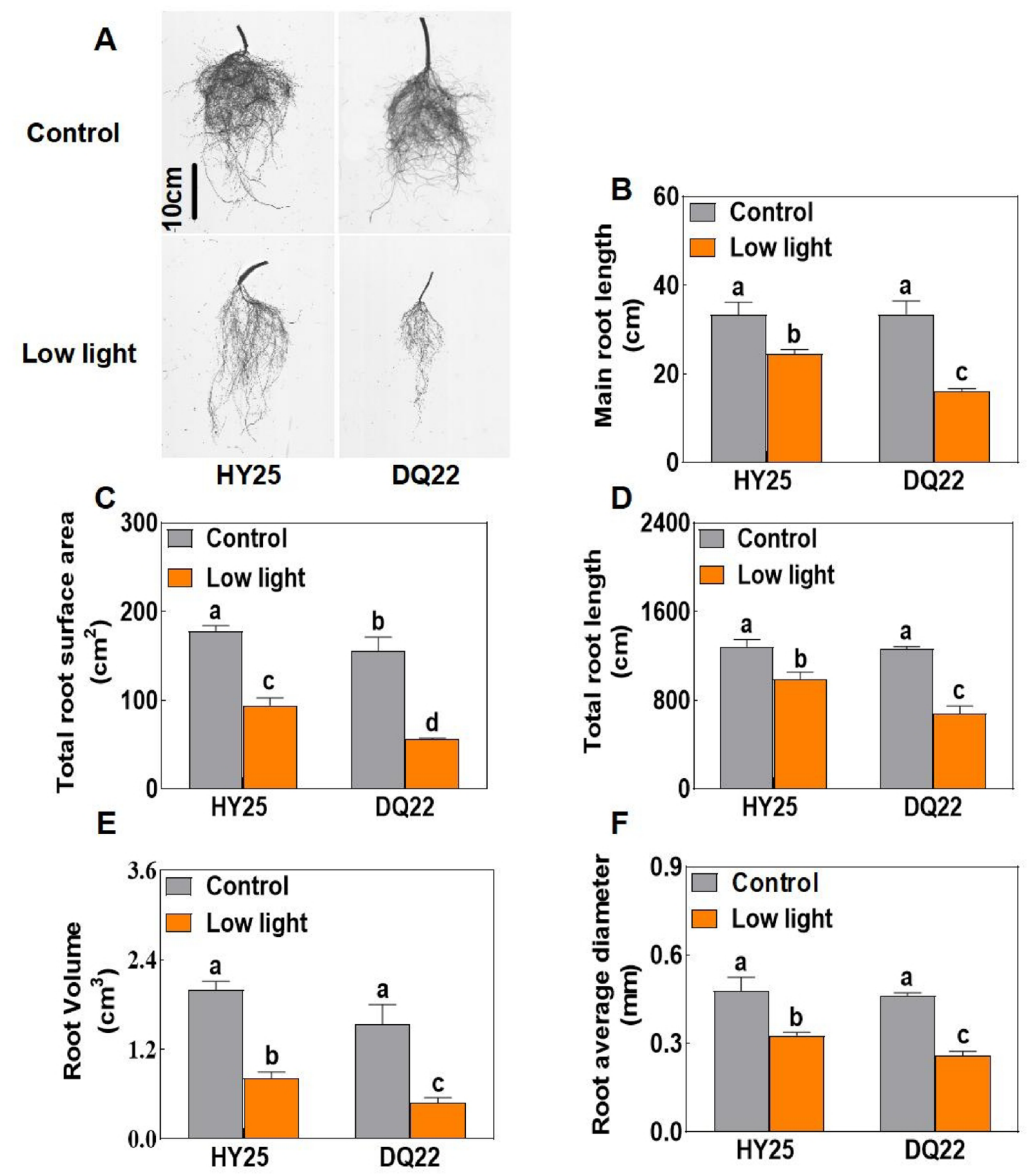
The seedling root phenotypes of watermelon lines with different tolerances under normal and low-light-stress conditions. The root phenotype (**A**), main root length (**B**), total root surface area (**C**), total root length (**D**), root volume (**E**), and average root diameter (**F**) of watermelon seedlings under normal light (control) and low-light-stress conditions at 25 days. HY25: tolerant line. DQ22: sensitive line. The values are the means ± standard deviations (*n* = 4). Different letters indicate significant differences (*P* < 0.05) according to Tukey’s test

We further evaluated the root growth phenotype of watermelon seedlings under normal and low-light conditions (Fig. 2). Under normal light conditions, the root systems of HY25 and DQ22 seedlings grew normally, and there were no significant differences in root growth indicators such as total root length, root volume, and root average diameter between the two lines (Fig. 2A). However, the low-light treatment significantly inhibited the growth and development of watermelon roots, with DQ22 plants experiencing the most severe inhibition, as evidenced by the reduction in watermelon root architecture traits, such as main root length (52.04%) (Fig. 2B), total root surface area (64.03%) (Fig. 2C), total root length (46.30%) (Fig. 2D), root volume (68.35%) (Fig. 2E), and average root diameter (43.92%) (Fig. 2F) formed per plant. Compared with that of the normal plants, the root growth index of the HY-25 plants decreased by 22.82 to 59.08% under low-light conditions (Fig. 2B–F).

### Analysis of chlorophyll fluorescence, photosynthetic capacity, chlorophyll content and relative electrolyte leakage in low-light tolerant of watermelon lines

**Fig. 3:**
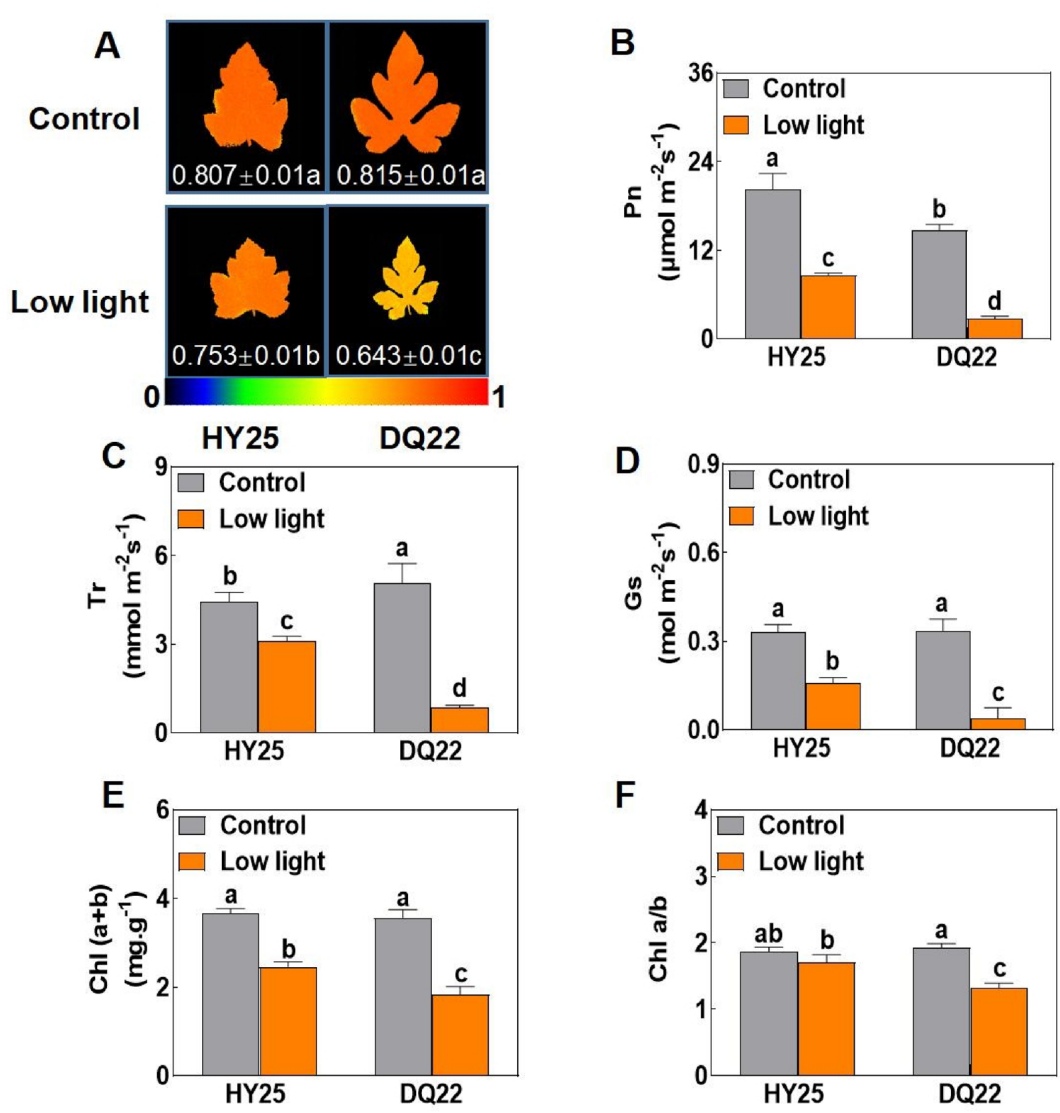
Analysis of the photosynthetic capacity of watermelon strains with different tolerances under normal and low-light stress. The maximal photochemical efficiency of PSII (Fv/Fm) in the leaves of watermelon seedlings under normal light (control) and low-light-stress conditions (**A**). Photosynthesis rate (Pn) (**B**), transpiration rate (Tr) (**C**), stomatal conductance (Gs) (**D**), chlorophyll content (chlorophyll *a + b*) (**E**), and chlorophyll content (chlorophyll *a/b*) (**F**) in the leaves of different watermelon lines under normal light (control) and low-light-stress conditions at 25 d. The values are the means ± standard deviations (*n* = 4). Different letters indicate significant differences (*P* < 0.05) according to Tukey’s test

To evaluate the impact of low-light stress on photosynthetic performance, we first measured the maximum photochemical efficiency of photosystem II (Fv/Fm) in the leaves of HY25 and DQ22 seedlings. Under low-light conditions, the Fv/Fm of DQ22 decreased significantly from 0.815 to 0.643, indicating a marked reduction in PSII efficiency. In contrast, the Fv/Fm of HY25 decreased only slightly, from 0.807 to 0.753, under the same conditions (Fig. 3A).

Furthermore, compared with those under normal light, the contents of Pn, Gs, and Tr in watermelon seedling leaves under low-light stress decreased to varying degrees. After the low-light treatment, the contents of the contents of Pn, Tr, and Gs in the leaves of the DQ22 line decreased by 81.32, 82.84, and 88.02%, respectively, and greater decreases of 57.38, 30.18, and 52.12%, respectively, in Pn, Gs, and Tr were detected in the HY25 line (Fig. 3B-D). This indicated a sharply decreased rate of photosynthate accumulation in the leaves of the sensitive line (DQ22).

Next, observations of chlorophyll (*Chl*) contents in the different watermelon inbred lines under low-light stress revealed that the changes in the total *Chl* content in the functional leaves under low-light stress were different. The content of *Chl (a + b)* in the leaves of the HY25 inbred line decreased by 32.79%, and the content of *Chl (a + b)* in the leaves of the DQ22 lines decreased extremely significantly by 48.53% (Fig. 3E, F). Under low-light stress, the ratio of chlorophyll *a* to *b (Chl a/b)* in the leaves of the sensitive line (DQ22) was 31.78% lower than that under normal-light conditions, and the *Chl a/b* of the tolerant line (HY25) did not substantially change under either light condition (Fig. 3E, F).

### Carbohydrate accumulation, soluble protein content, proline content changes in different watermelon lines under low-light stress

**Fig. 4:**
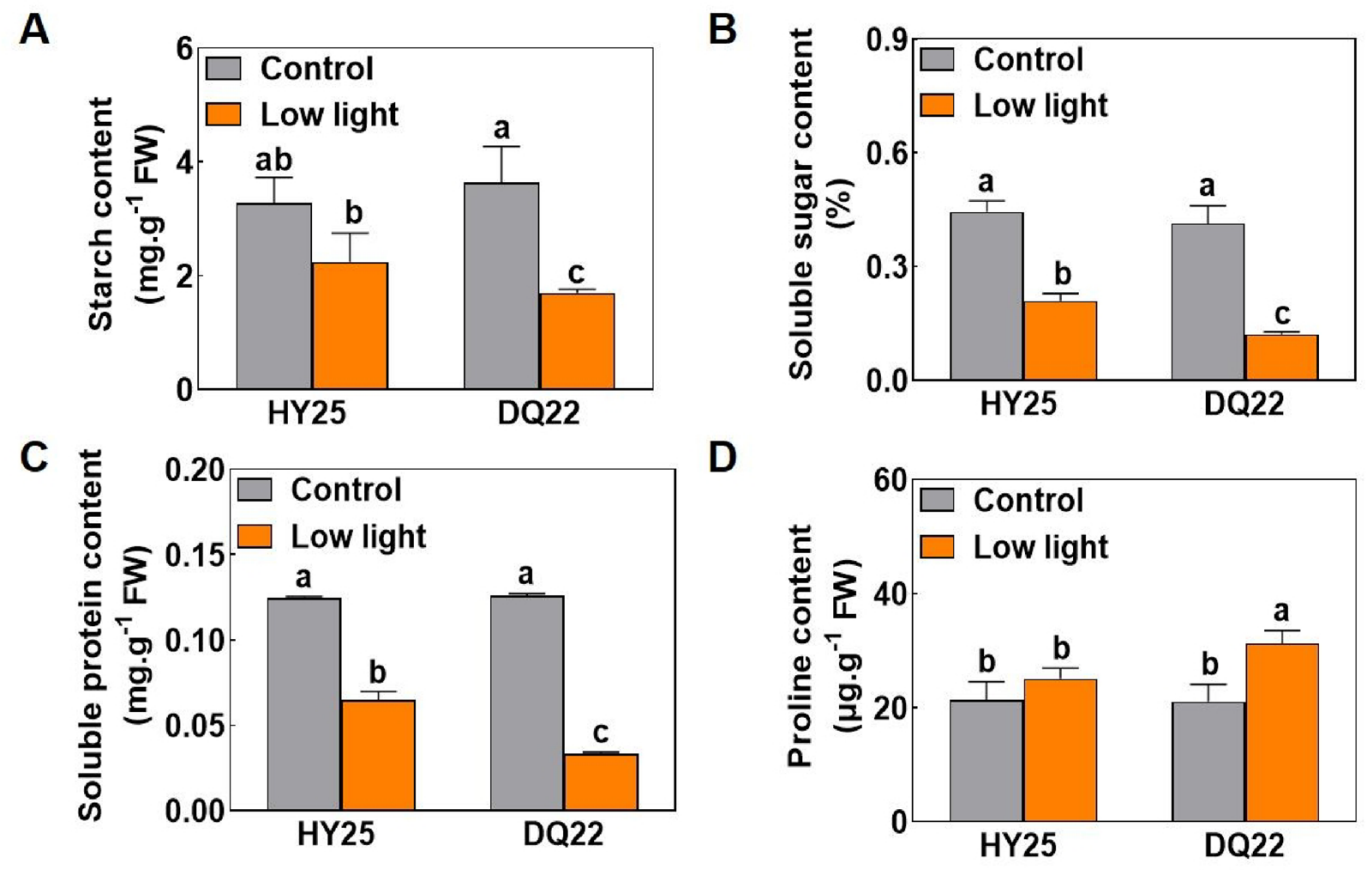
Effects of low-light stress on starch, soluble protein, and proline contents in the leaves of watermelon lines. The contents of starch (**A**), soluble sucrose (**B**), soluble protein (**C**), and proline (**D**) were measured in the leaves of HY25 (low-light-tolerant) and DQ22 (low-light-sensitive) seedlings under normal light (control) and low-light conditions after 25 days of treatment. The values are the means ± standard deviations (*n* = 4). Different letters indicate significant differences (*P* < 0.05) according to Tukey’s test

Compared with those under normal light, the contents of starch and sucrose in watermelon seedling leaves under low-light stress decreased to varying degrees (Fig. 4). After the low-light treatment, the contents of starch and sucrose in leaves of the HY25 line decreased by 31.64 and 53.03%, respectively, and larger decreases of 53.30% and 70.59% in starch and sucrose contents, respectively, were detected in DQ22, indicating a sharply decreased rate of carbohydrate accumulation in the leaves of the sensitive line (DQ22) (Fig. 4A, B). Moreover, under normal light conditions, there were no significant differences in the soluble protein content between the two lines. However, under low-light stress, the soluble protein contents in the leaves of the sensitive line (DQ22) and the tolerant line (HY25) were 73.52 and 47.94% lower than under normal-light conditions, respectively (Fig. 4C). In addition, under low-light stress, proline content in the leaves of the sensitive line (DQ22) was 32.49% higher than that under normal-light conditions, and the proline content of the tolerant line (HY25) did not substantially change under either light condition (Fig. 4D).

### Malondialdehyde content (MDA), electrolyte leakage, ROS accumulation, and antioxidant responses under low-light stress

**Fig. 5:**
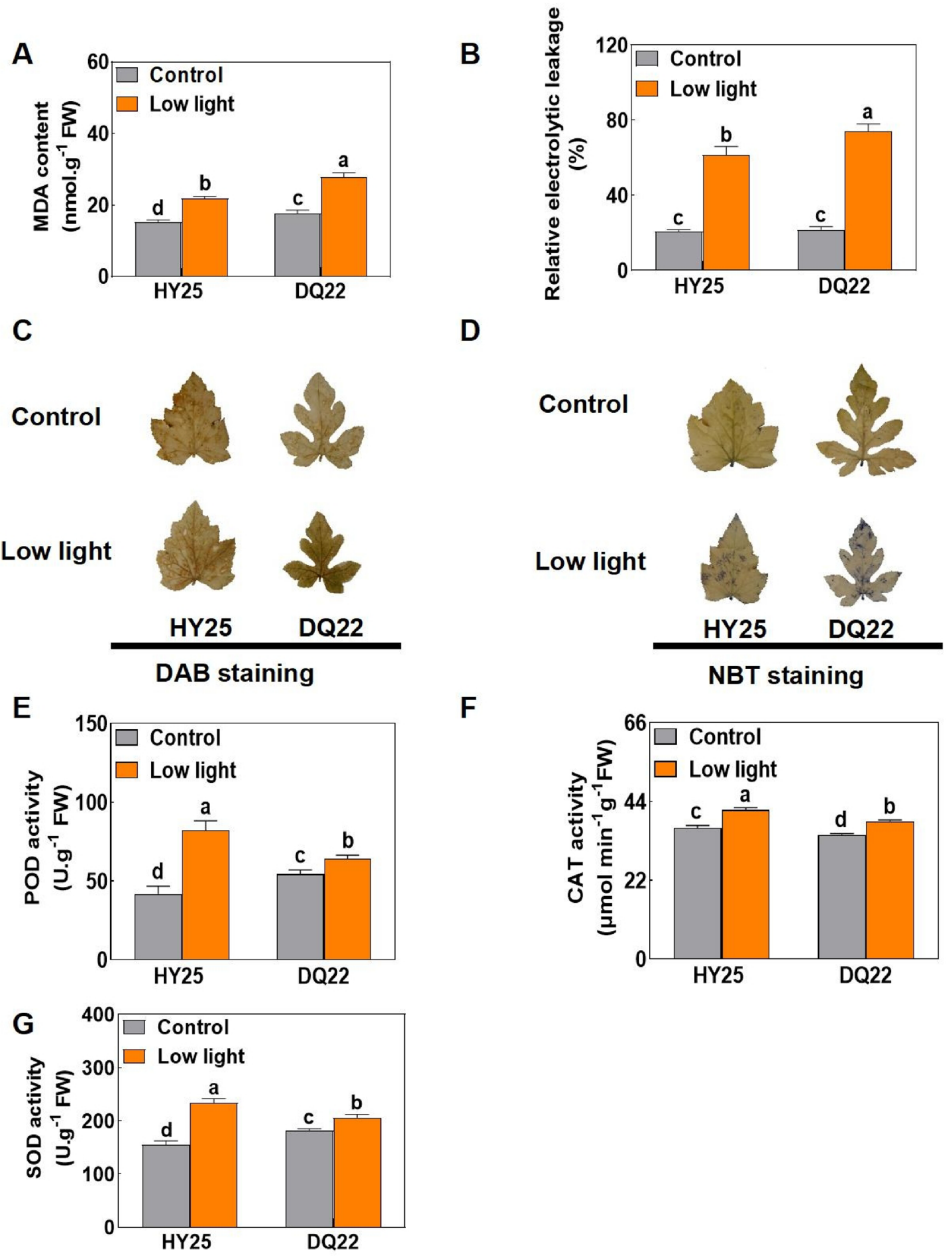
Effects on the activity of antioxidant enzymes, the MDA content and the REL content in the leaves of watermelon strains with different tolerances under normal and low-light stress. The MDA content (**A**), REL content (**B**), DAB (**C**) and NBT staining (**D**) and activities of antioxidant defence enzymes such as POD (**E**), CAT (**F**), and SOD (**G**) in the leaves of watermelon seedlings under normal light (control) and low-light stress conditions at 25 d. The values are the means ± standard deviations (*n* = 4). Different letters indicate significant differences (*P* < 0.05) according to Tukey’s test

The results indicated that under normal light conditions, there were no significant differences in the MDA content or REL between the watermelon seedlings of HY25 and DQ22 (Fig. 5A, B). However, compared with those under normal light, the MDA content and REL in the leaves of watermelon seedlings under low-light stress increased to varying degrees (Fig. 5A, B). After low-light treatment, the MDA content and REL in the leaves of the HY25 line increased by 43.14 and 195.85%, respectively, and the MDA content and REL content of DQ22 increased more significantly by 58.27 and 240.70%, respectively (Fig. 5A, B), indicating that the DQ22 plants were more severely damaged by low light.

To assess the accumulation of reactive oxygen species (ROS), including hydrogen peroxide (H₂O₂) and superoxide radicals (O₂·⁻), in response to low-light stress, watermelon leaves from both the HY25 (tolerant) and DQ22 (sensitive) lines were subjected to histochemical staining using 3,3′-diaminobenzidine (DAB) and nitroblue tetrazolium (NBT). Under normal light conditions, leaves from both lines presented minimal staining, indicating low basal ROS levels (Fig. 5C, D). However, under low-light stress, treated leaves presented pronounced dark brown (DAB, indicating H₂O₂) and navy blue (NBT, indicating O₂·⁻) staining, with DQ 22 leaves showing significantly more intense staining than HY25 leaves did (Fig. 5C, D). Notably, compared with the DQ22 plants, the HY25 plants presented increased peroxidase (POD), catalase (CAT), and superoxide dismutase (SOD) activities after low-light stress. Taken together, these results indicate that low light can induce the accumulation of ROS and that HY25, by enhancing its ability to scavenge ROS and induce an antioxidant defence system, increases its ability to respond to low-light stress (Fig. 5E–G).

### RNA-seq analysis and identification of differentially expressed genes

**Fig. 6:**
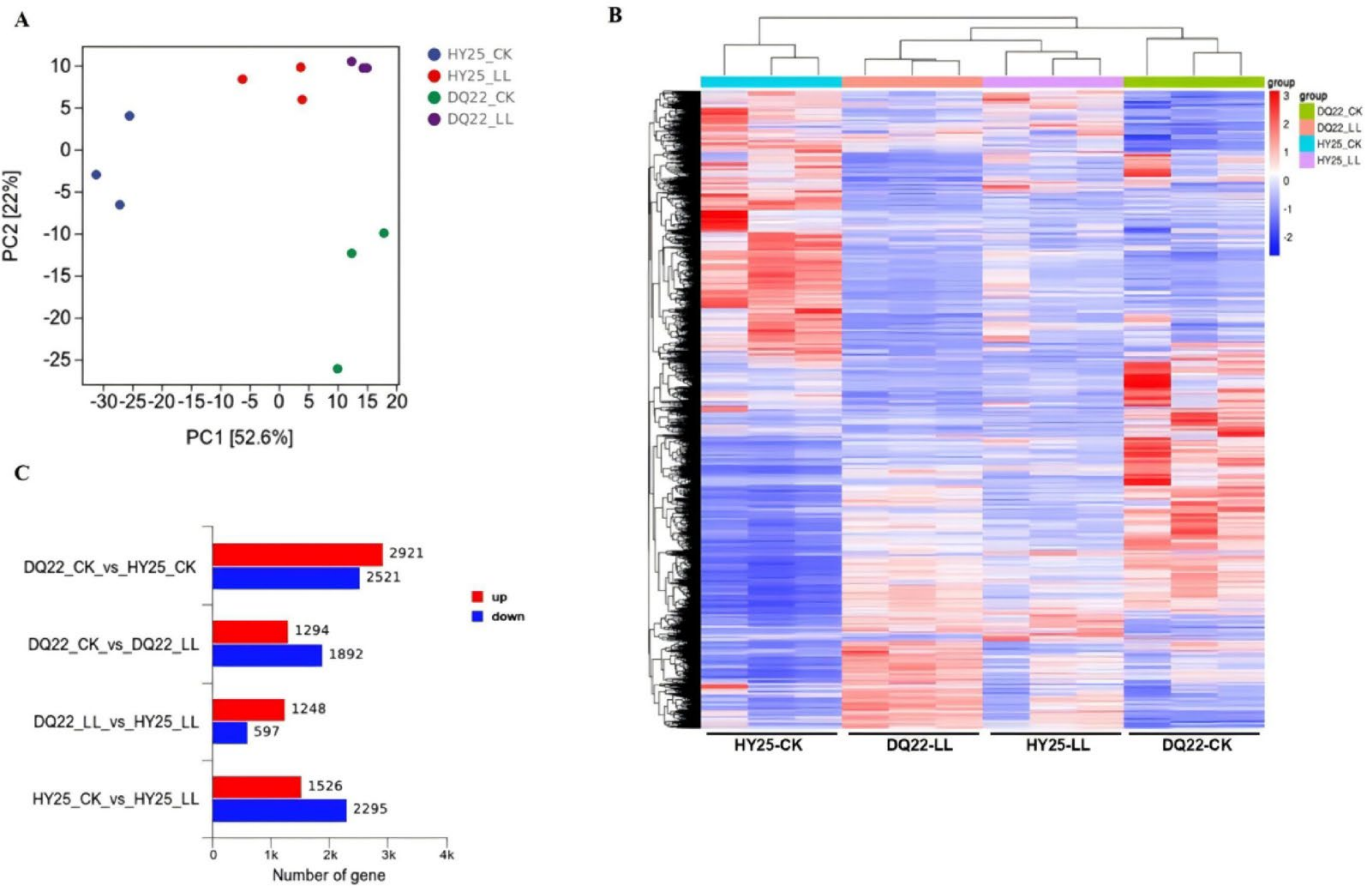
Transcriptomic analysis of differentially expressed genes (DEGs) in watermelon under low-light stress. Principal component analysis (PCA) of all 12 transcriptomes, showing distinct clustering patterns between treatment groups (**A**). Two-way hierarchical clustering of DEGs (fold change > 1.5, *p* < 0.05) across comparisons (**B**), with columns representing experimental groups and rows representing individual genes. Quantitative summary of DEGs identified in pairwise comparisons between genotypes (HY25 vs. DQ22) and light treatments (control vs. low-light treatment) (**C**). *HY25-CK* tolerant line HY25 under normal light, *DQ22-CK* sensitive line DQ22 under normal light, *HY25-LL* tolerant line HY25 under low light, *DQ22-LL* sensitive line DQ22 under low light

To explore the genes differentially expressed in response to low light compared with the control conditions in both inbred lines (HY25 and DQ22), we performed RNA-seq on leaves (48 h after low-light treatment and 48 h after normal light treatment as a control). As shown in Supplemental Table S2, the total mapping ratio of filtered clean reads in each sample ranged from 97.93 to 98.10%. The Q20 and Q30 values of all 12 samples were greater than or equal to 97.56 and 93.2%, respectively. Moreover, principal component analysis (PCA) of all 12 transcriptomes revealed that the first (PC1) and second (PC2) variances were 52.6 and 22%, respectively, and three biological replicates from each sample were clustered together (Fig. 6A). We identified 8,291 differentially expressed genes (DEGs; fold change > 1.5, *p* < 0.05) across all comparisons, with tight clustering of biological replicates in the heatmap confirming the high reproducibility of the expression patterns (Fig. 6B; Supplemental Table S3). Differential expression analysis revealed 5,442 DEGs between HY25 and DQ22 under normal light (2,921 upregulated/2,521 downregulated), 1,845 DEGs between lines under low light (1,248 upregulated/597 downregulated), 3,186 DEGs in DQ22 across light conditions (1,294 upregulated/1,892 downregulated), and 3,821 DEGs in HY25 across conditions (1,526 upregulated/2,295 downregulated) (Fig. 6C). The strong correlation (*r > 0.87*) between the RNA-seq and qRT‒PCR results for the fourteen selected DEGs validated the reliability of the dataset (Fig. S1).

### Transcriptome and photosynthesis-related metabolic pathway analysis of watermelon leaves under low-light stress

**Fig. 7:**
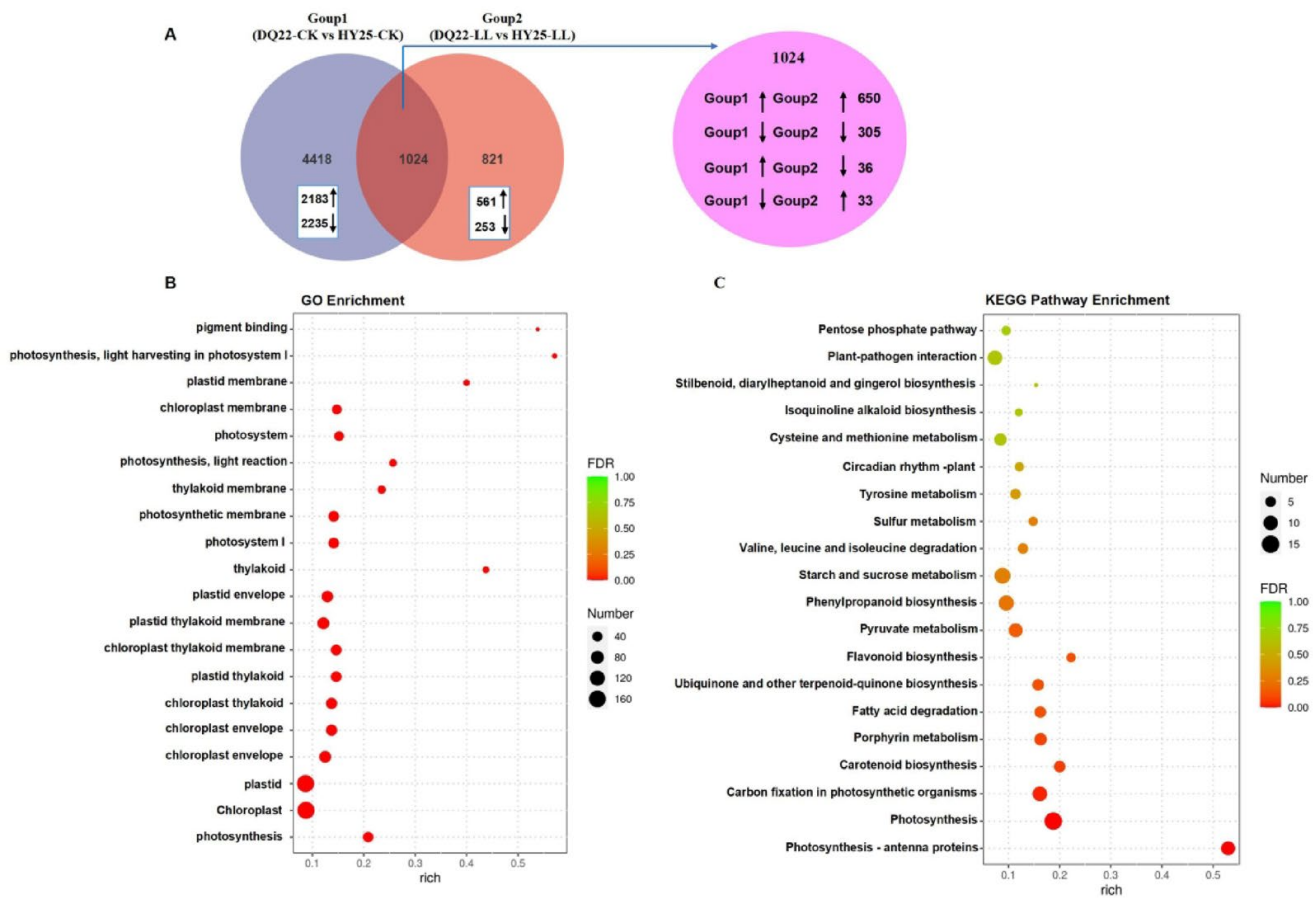
Transcriptome analysis of watermelon leaves of the HY25 and DQ22 lines under low-light stress. Venn diagram of DEGs under low-light stress (**A**). GO enrichment analysis results of 1024 DEGs with a fold change > 1.5 (**B**). KEGG enrichment of 1024 DEGs with a fold change > 1.5 (**C**). HY25-CK: tolerant line HY25 under normal light. DQ22-CK: sensitive line DQ22 under normal light. HY25-LL: tolerant line HY25 under low light. DQ22-LL: sensitive line DQ22 under low light. Group 1: DQ22-CK vs. HY25-CK. Group 2: DQ22-LL vs. HY25-LL. The arrows represent the changes in 1024 differentially expressed genes (DEGs) identified in both lines (DQ22 and HY25) under the control and low-light conditions (Group 1 vs. Group 2)

**Fig. 8:**
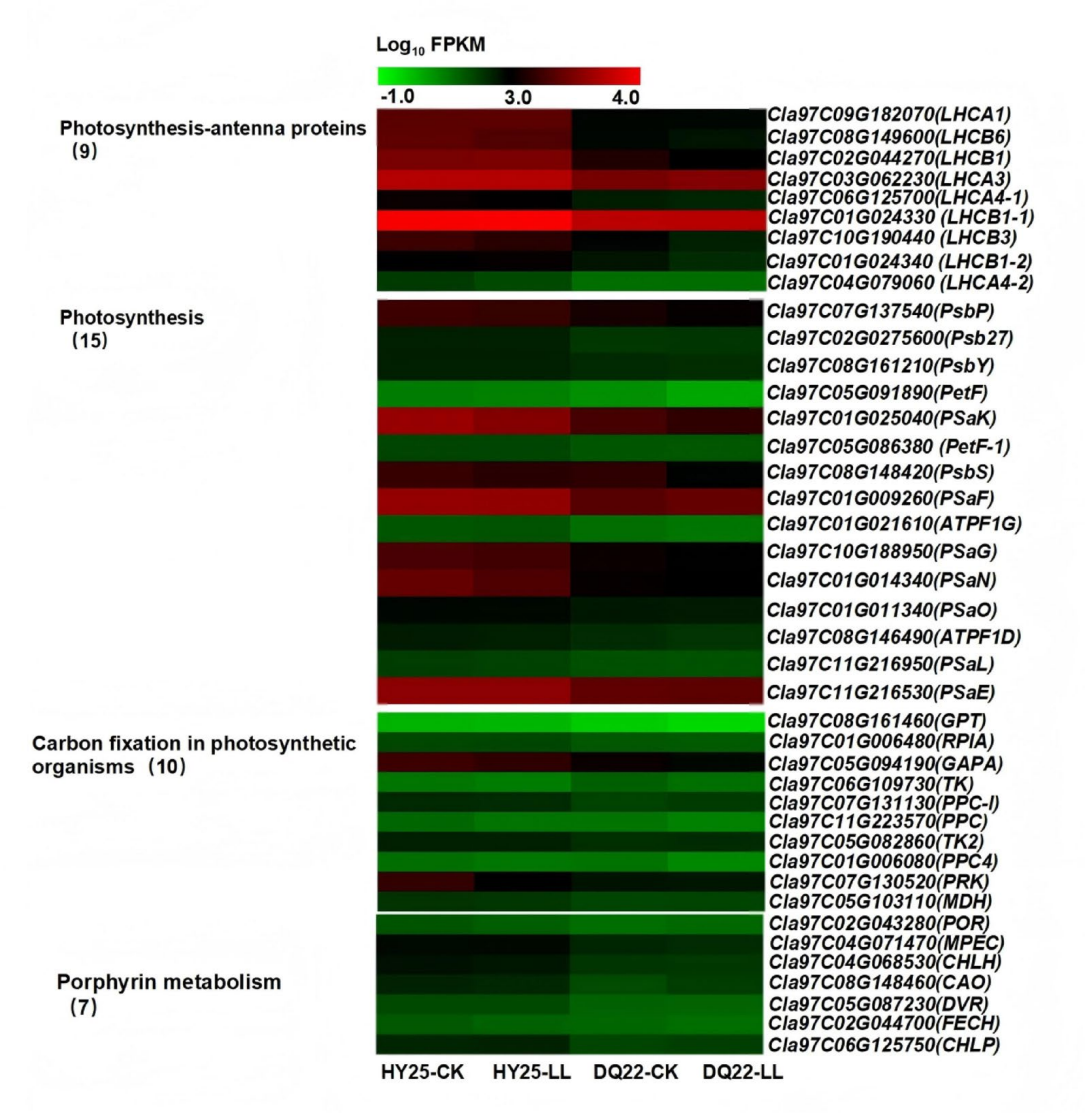
Pathway analysis of photosynthesis-antenna proteins, photosynthesis, carbon fixation in photosynthetic organisms and porphyrin metabolism in leaves of watermelon strains with different tolerances under normal and low-light stress

Further analysis of these datasets revealed that the remaining 4418 genes were differentially expressed (2183 upregulated and 2235 downregulated genes) in the leaves between DQ22 and HY25 under the normal light conditions (Group 1, DQ22-CK vs. HY25-CK), and a total of 821 genes were differentially expressed (561 upregulated and 253 downregulated genes) in the leaves of DQ22 and HY25 under low-light conditions (Group 2, DQ22-LL vs. HY-25- LL) (Fig. 7A). A total of 1024 differentially expressed genes (DEGs) were identified in both lines under both the control and low-light conditions (Group 1 vs. Group 2), namely, 650 co-upregulated genes, 305 codownregulated genes, 36 genes upregulated in Group 1 and downregulated in Group 2, and 33 genes downregulated in Group 1 and upregulated in Group 2 (Fig. 7A). Gene Ontology (GO) analysis revealed that these 1024 genes were enriched mainly in photosynthesis, chloroplasts, plastids, the chloroplast membrane, the photosystem, light harvesting proteins and other photosynthesis-related pathways, among which the DEGs enriched in the plastid and chloroplast pathways were the most abundant (Fig. 7B). Furthermore, Kyoto Encyclopedia of Genes and Genomes (KEGG) analysis of these 1024 DEGs revealed that the photosynthesis and photosynthesis-antenna protein pathways were significantly enriched (Fig. 7C). Therefore, we selected forty-one genes related to photosynthesis enriched in the leaves, which were differentially expressed under low-light stress in the differently tolerant lines DQ22 and HY25. Moreover, KEGG pathway analysis of photosynthesis revealed that photosynthesis-antenna proteins, photosynthesis, carbon fixation in photosynthetic organisms and porphyrin metabolism were affected differently in both lines after low-light treatment (Fig. 8).

In the low-light-tolerant line HY25, low-light treatment induced upregulation of key light-harvesting complex (LHC) genes (*LHCA1*, *LHCB1*, *LHCA3*, *LHCB1-1*, and *LHCB1-2*), while downregulating others (*LHCB6*, *LHCA4*, *LHCB3*, and *LHCB4*) (Fig. 8, Fig. S2A, and Supplementary Table 4).In contrast, the low-light-sensitive line DQ22 showed significant downregulation of most LHC genes under identical treatment conditions, with only *HLCA3* and *HLCA4-2* exhibiting marginal upregulation (Fig. 8, Fig. S2A, and Supplementary Table 4). Furthermore, some differentially expressed genes were detected by analysis of the photosynthesis pathway, such as genes involved in the pathway of photosystem II (PSII) (*PsbP*, *Psb27*,* PsbY* and *PsbS*), some genes involved in the in the pathway of photosystem I (PSI) (*PSaE*, *PSaF*, *PSaG*, *PSaK*, *PSaL*, *PSaO*, and *PSaN*), the *PetF* gene of photosynthethic electron transport, and some genes involved in the pathway of F-type ATPase (*ATPF1G and ATPF1*). The results revealed that the genes *ATPF1G* and *PSaE* were upregulated in the tolerant line (HY25) after low-light treatment, whereas the other photosynthesis pathway-related genes were downregulated. However, most of the genes were downregulated in the sensitive line (DQ22) except the *PSaF gene* (Fig. 8, Fig. S2B, and Supplementary Table 4). Notably, the expression levels of photosynthesis-related genes in the tolerant line (HY25) were significantly greater than in the sensitive line (DQ22) under both the control and low-light treatments (Fig. 8, Fig. S2B, and Supplementary Table 4). In addition, genes involved in carbon fixation in photosynthetic organisms and porphyrin metabolism were analysed. The results revealed that, compared with those in the sensitive line (DQ22), the expression of most genes involved in carbon fixation in photosynthetic organisms and porphyrin metabolism in the tolerant line (HY25) was significantly greater, regardless of the control or low-light treatment (Fig. 8 and Supplementary Table 5).

**Fig. 9:**
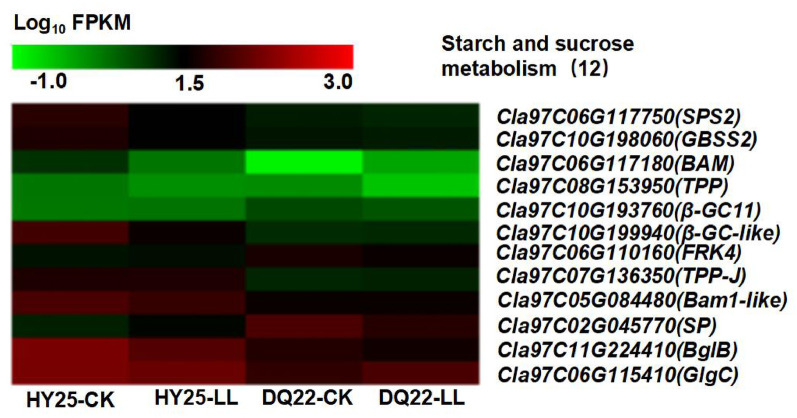
Low-light stress-induced expression patterns of differentially expressed genes (DEGs) involved in the metabolic pathways of starch and sucrose

KEGG pathway analysis of polysaccharides revealed that the expressions of starch and sucrose metabolism-associated genes were dramatically different in watermelon leaves under low-light stress than in those under normal-light conditions. A total of 12 genes related to the starch and sugar metabolism pathway were significantly differentially expressed. Of these genes, 9 were downregulated, and 3 were upregulated in HY25 leaves after low-light stress; however, only 10 genes were differentially expressed in DQ22 (4 upregulated and 6 downregulated) (Fig. 9 and Supplementary Table 6). Notably, the expression levels of starch and sugar metabolism-related genes except the *β-GC*, *FPK4*, and *SP*, in the tolerant line (HY25) were significantly greater than those in the sensitive line (DQ22), regardless of the light treatment (Fig. 9 and Supplementary Table 6). These genes included the starch synthase genes GBSS2 and Glgc/AGPase. QRT‒PCR validation of the 4 selected genes yielded consistent results (Fig. S3). ADP glucose pyrophosphorylase (Glgc/AGPase) is responsible for catalysing glucose 1-phosphate to produce ADP glucose (the direct precursor of starch synthesis), which is the rate-limiting step of starch synthesis. GBSS, a granule-bound starch synthase, specifically catalyses the synthesis of amylose and binds with starch granules. The beta-amylase gene Bam1 is required for starch breakdown. Sucrose phosphate synthase (SPS2) catalyses the key step of sucrose biosynthesis, combines uridine diphosphate glucose (UDPG) with fructose-6-phosphate to form sucrose-6-phosphate and finally forms sucrose, which plays a central role in the sucrose synthesis pathway and directly affects the transportation and distribution of sugars in plants (Fig. 9 and Supplementary Table 6).

### Low light stress induced the expression of auxin response differentially expressed genes (DEGs)

**Fig. 10:**
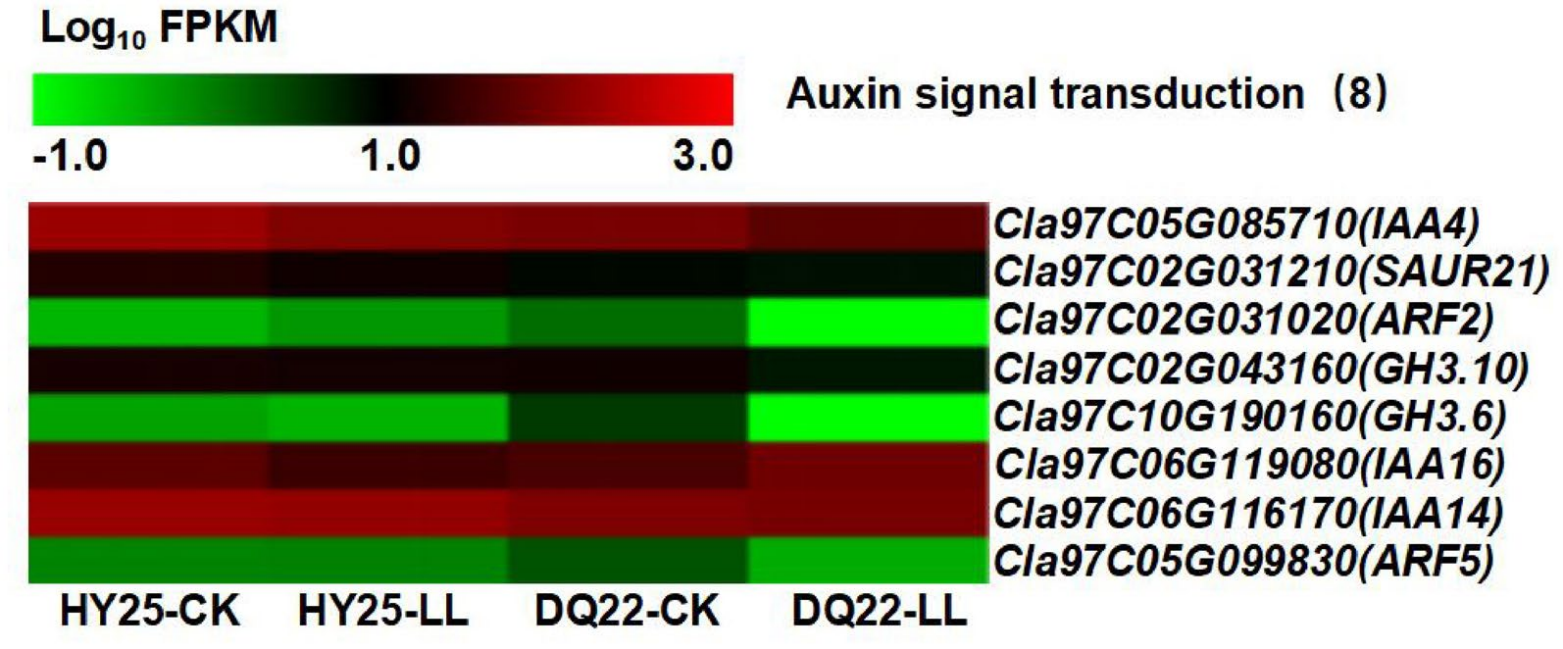
Low-light stress differentially induces the expressions of auxin-responsive differentially expressed genes (DEGs)

KEGG pathway analysis of auxin metabolism revealed that the expressions of auxin metabolism-related genes in watermelon leaves under low-light stress significantly differed from those under normal-light conditions. The expressions of 8 genes related to the auxin metabolic pathway significantly differed; among these genes, 6 were downregulated, and 2 were upregulated in HY25 leaves under low-light stress (Fig. 10 and Supplementary Table 7). However, 7 genes were downregulated, and 1 gene was upregulated in DQ22 leaves under low light stress. QRT‒PCR analysis of the selected genes corroborated the transcriptomic findings (Fig. S4). Notably, the expressions of auxin metabolism-related genes in the resistant line (HY25) were significantly greater than in the sensitive line (DQ22), both for the control and low-light treatment (Fig. 10 and Supplementary Table 7).

## Discussion

The present study elucidated the differential responses of watermelon lines to low-light stress, providing novel insights into the physiological and molecular mechanisms underlying low-light adaptation in watermelon. As a photophilic crop, watermelon requires a high light intensity for optimal growth and fruit development. However, low-light stress in protected cultivation systems, particularly in regions with prolonged overcast conditions, severely limits productivity by disrupting morphological, photosynthetic, and metabolic processes. Our findings highlight the critical role of photosynthetic efficiency, carbon metabolism, auxin metabolism, and transcriptional regulation in conferring tolerance to low-light conditions, providing a theoretical basis for clarifying the mechanism of watermelon tolerance to low light and cultivating varieties with low-light tolerance.

### Low-light stress pressure and morphological responses of different watermelon lines tolerant to low-light stress

Low-light stress has adverse effects on plant growth and development, severely impairing several metabolic activities [10]. Under continuous low-light stress, the hypocotyls of plants become longer, the leaves yellow, the leaf area decreases, the main axis terminates, and the meristem at the top of the stem turns into flowers [41]. Plants with different traits respond differently to low-light conditions. Compared with DQ22, HY25 presented superior growth performance under low-light conditions, with minimal reductions in plant height, stem diameter, leaf length, petiole length, whole fresh weight, whole dry weight and root growth indices. These findings align with those of previous studies showing that low-light-tolerant plants often maintain structural integrity to optimize light capture and resource allocation under suboptimal conditions [42]. The pronounced dwarfism, leaf yellowing, and root inhibition observed in DQ22 are consistent with reports in other crops, where low-light-sensitive genotypes exhibit impaired biomass accumulation due to disrupted source‒sink relationships [17, 43–45].

### Photosynthesis ability and metabolic accumulation responses of watermelon lines under low-light stress

Photosynthesis, which plays a crucial role in plant growth and development, provides necessary materials and energy for plants and is the basis of crop biomass and yield [46, 47]. A previous study suggested that light limitation or shading stress decreases carbon fixation and the canopy net photosynthetic rate and has a negative effect on photosynthesis [48, 49]. Consistent with previous studies, we also found that low-light stress significantly inhibited the photosynthetic performance of watermelon seedlings, and this inhibition was more obvious in the sensitive strain DQ22. Compared with those under normal light, the photosynthetic parameters Fv/Fm, Pn, Tr and Gs of DQ22 seedling leaves under low light decreased sharply, ranging from 22.97 to 88.02%. However, HY25, a low-light-tolerant line, has the ability to maintain photosynthetic capacity, which is supported by higher values of Fv/Fm, Pn, Gs and Tr and indicating that photochemical efficiency and stomatal regulation are increased; these are critical for balancing energy acquisition and carbon assimilation in light-limited environments [20].

Low-light environments interfere with the normal photosynthetic activity of plants and reduce the accumulation of photosynthates by affecting starch and sucrose metabolism in the carbon source [50, 51]. The differences in carbohydrate dynamics between HY25 and DQ22 underscore the metabolic basis of low-light tolerance. The sharp decline in starch and sucrose in the sensitive line DQ22 mirrors the findings of studies where low-light-sensitive plants suffer from carbon starvation due to insufficient photosynthate production [52]. The ability of HY25 to maintain relatively high soluble sugar and protein levels, suggests enhanced carbon partitioning and metabolic flexibility, possibly through alternative substrate utilization or reduced catabolic activity.

In addition, previous studies have indicated that the *Chl* content and the ratio of chlorophyll a to b (*Chl a/b*) in leaves dynamically adapt to variations in light intensity to a certain degree, in theory determining the potential grain yield of plants [53, 54]. We also found that the *Chl* content and the *Chl a/b* ratio in the leaves of the sensitive line (DQ22) under low-light stress were lower than those under normal light conditions and that the *Chl a/b* ratio of the tolerant line (HY25) did not substantially change under either light condition. This may be due to the severe damage to chloroplasts caused by low-light stress in the sensitive line, resulting in a sharp decrease in the *Chl* content. The decline in chlorophyll content and altered *Chl a/b* ratio in DQ22 further reflect its susceptibility to low-light stress. Chlorophyll *a/b* ratios are tightly linked to the composition of light-harvesting complexes (LHCs), and their reduction under low light may indicate a failure to adjust antenna size, leading to excess excitation pressure and photooxidative damage [55]. In contrast, the stable chlorophyll profile of HY25 implies efficient modulation of LHCs to optimize light utilization, a trait observed in shade-tolerant species [27].

### Response of different watermelon lines to low-light stress in terms of ROS accumulation and the antioxidant defense system

Low-light stress not only induces growth inhibition in plants but also causes various physiological dysfunctions, such as reduced photosynthetic efficiency, inhibited biological carbon fixation, accumulation of ROS, and membrane lipid peroxidation [56–59]. In this study, we found that under low-light stress, the sensitive line DQ22 exhibited significant ROS accumulation in the leaves, leading to membrane lipid peroxidation, with a marked increase in the content of malondialdehyde (MDA) content, damage to the cell membrane structure, leakage of intracellular electrolytes, and an increase in REL. In contrast, the low light-tolerant variety HY25 presented a lower accumulation rate of MDA under low-light stress, lower REL, less ROS accumulation, and higher activities of antioxidant enzymes such as peroxidase (POD), catalase (CAT), and superoxide dismutase (SOD) in the leaves. These findings indicate that the tolerant variety can more effectively remove ROS and reduce membrane lipid peroxidation damage in the membrane.

The decrease in photochemical efficiency is also related to lipid peroxidation in the membrane. Low-light stress often leads to oxidative damage, which manifests as the generation of reactive oxygen species (ROS). As toxic substances, ROS act as signalling molecules to regulate abiotic stress effects in plants. Abiotic stress can disturb homeostasis in the cell and rapidly induce the production of a large amount of ROS through aerobic metabolism, thereby triggering oxidative stress, membrane lipid peroxidation and metabolic disorders [60]. To maintain redox balance, plants usually quench ROS with the help of antioxidant enzymes and nonenzymatic systems. Antioxidant enzymes and other antioxidants are important components for eliminating ROS under abiotic stress [61, 62].

### Transcriptome analysis and expression of photosynthesis-related genes in watermelon lines under low-light tolerance

Photosynthesis is dramatically affected by low-light stress, and photosynthesis ability and photosynthate accumulation decrease under stress, resulting in reduced expression levels of genes involved in photosynthesis [63, 64]. Earlier results revealed that when the photosystem is damaged under stress conditions, PSII repair and *Chl* turnover subsequently occur, and more PSII genes and genes encoding LHC components are downregulated [65]. The results revealed significant differences in gene expression related to photosynthesis-antenna proteins, photosynthesis, carbon fixation and the porphyrin metabolism pathway during low-light adaptation. Some genes related to the photosynthesis pathway were expressed at relatively high levels in the leaves of the low-light-tolerant line HY25 but expressed at relatively low levels in the leaves of the sensitive line DQ22. DQ22 exhibited downregulation of key light-harvesting chlorophyll protein complex (LHC) genes (e.g., *LHCA1*, *LHCA4-1*, *LHCB1*, *LHCB1-1*, *LHCB1-2*,* LHCB3*, and *LHCB6*) under low-light stress, suggesting impaired chlorophyll antenna assembly and reduced light-energy capture efficiency. In contrast, HY25 maintained upregulated expression of the light-harvesting chlorophyll protein complex (LHC) genes *LHCA1*, *LHCB1*, *LHCA3*, *LHCB1-1*, and *LHCB1-2*, potentially preserving photosynthetic efficiency under stress. This divergence aligns with the observed phenotypic tolerance of the two watermelon plant types under low-light conditions. Similar results have also been reported in cucumber [9]and rice [10]. This may be one of the main reasons why the low-light-tolerant line was more tolerant to low-light stress.

Researchers have also revealed that the altered structures of the stomata, chloroplast lamellae and thylakoids under low-light stress resulted in a decreased concentration of CO_2_ and rate of electron transfer in chloroplasts, thereby decreasing photosynthate accumulation capability, which might explain the decrease in the number of starch grains in rice [66]. Our study revealed that the tolerant HY25 plants maintained relatively high carbohydrate production levels by maintaining an efficient Pn even under low light, which, in turn, was achieved by maintaining relatively high levels of *Chl* content, starch and sucrose metabolism-related gene expression (such as *AGPase*, *GBSS2*, *BglB*, *SP2*, *TPP*, etc.) compared with those in the low-light-sensitive line DQ22. ADP glucose pyrophosphorylase (Glgc/AGPase) is responsible for catalysing glucose 1-phosphate to produce ADP glucose (the direct precursor of starch synthesis), which is the rate-limiting step of starch synthesis. GBSS, a granule-bound starch synthase, specifically catalyses amylose synthesis and binds with starch granules. Sucrose phosphate synthase (SPS2) catalyses the key step of sucrose biosynthesis; it combines uridine diphosphate glucose (UDPG) with fructose-6-phosphate to form sucrose-6-phosphate and finally forms sucrose, which plays a central role in the sucrose synthesis pathway and directly affects the transportation and distribution of sugars in plants. Trehalose 6-phosphate phosphatase (TPP) is involved mainly in the trehalose metabolic pathway and produces trehalose through the dephosphorylation of trehalose 6-phosphate. Trehalose and its precursors act as signalling molecules in plants, regulating carbon allocation, the stress response, and growth and development. These results implied that the photosynthetic ability and glycosylation of the sensitive line were relatively weak under low-light stress, likely caused by destruction of the leaf structure and chloroplast ultrastructure [67].

### Transcriptome analysis and expression of auxin-related genes in watermelon lines with different low-light tolerances

According to Gaba and Black, plant hormones can simulate light to regulate plant growth and development, indicating that hormones may be the second messengers of light signal transmission [68]. Studies have shown that exogenous GR24 might alleviate low-light stress-induced growth inhibition by regulating the assimilation of carbon and antioxidants and the endogenous strigolactone content, thereby increasing the tolerance of cucumber seedlings to low-light stress [69]. Therefore, the use of plant growth regulators to improve the adaptability of plants to low light is effective [25]. Our research revealed that the tolerance of HY25 line is achieved by regulating the expression levels of auxin-related genes under low-light conditions to levels significantly greater than those in the sensitive line (DQ22). This divergence aligns with the observed phenotypic tolerance of the two watermelon plant types under low-light conditions.

## Conclusion

This study demonstrated that low-light stress significantly inhibits watermelon growth and physiological metabolism, primarily by impacting the photosynthetic capacity and photosynthate accumulation. The observed effects were mediated by structural alterations in leaves and chloroplasts, ROS accumulation, antioxidant activity modulation, and differential expression of photosynthesis-related genes, with distinct responses between tolerant (HY25) and sensitive (DQ22) lines. The sensitive line exhibited severe photosynthetic impairment, characterized by a reduced chlorophyll content and significant downregulation of photosynthesis-related genes, leading to markedly decreased photosynthate accumulation. In contrast, the tolerant line maintained relatively stable photosynthetic performance and gene expression levels under stress conditions. Transcriptomic analysis further revealed differential expression of genes involved in photosynthesis, carbohydrate metabolism (starch and sucrose), and auxin pathways between the two genotypes. By comparing phenotypic and molecular responses to low-light stress, this study identified critical adaptation periods and response characteristics in watermelon. These findings provide a foundation for future functional studies of low-light responsive genes and will facilitate the development of molecular markers for early screening of low-light-tolerant germplasms, ultimately supporting breeding programs aimed at improving watermelon productivity under suboptimal light conditions. While this study focused on semilethal low-light conditions, future work should investigate gradual acclimation responses and field-based validation under fluctuating light regimes. Integrating multiomics approaches (e.g., proteomics and metabolomics) could further elucidate the posttranscriptional and metabolic networks governing low-light adaptation.

## Supplementary Information

Below is the link to the electronic supplementary material.

Supplementary Material 1

Supplementary Material 2

Supplementary Material 3

Supplementary Material 4

Supplementary Material 5

## Data Availability

The raw data of RNA-seq has been deposited in the NCBI repository, accession number PRJNA1260060, and the data that supports the results of this study can be found within the manuscript and its supplementary materials.
