# Supplementary figures and images for: Integrative analysis of different low-light-tolerant watermelon lines in response to low-light stress
Source: BMC Plant Biol. 2025 Aug 21;25:1107. doi: 10.1186/s12870-025-07180-8 (PMC12369212; doi:10.1186/s12870-025-07180-8)

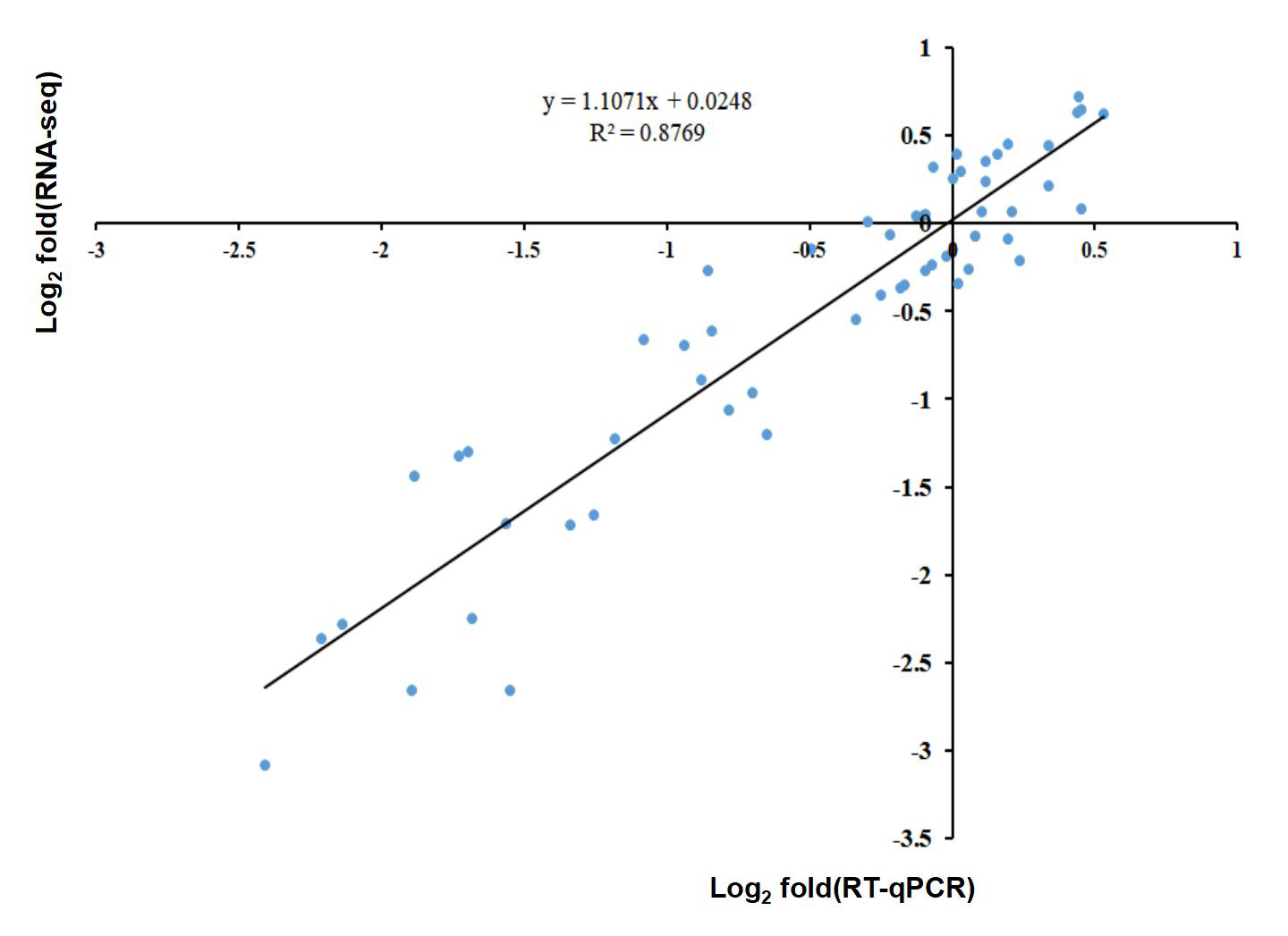

Supplement: Supplementary file 1 — Supplementary Material 1 [file 12870_2025_7180_MOESM1_ESM.jpg]

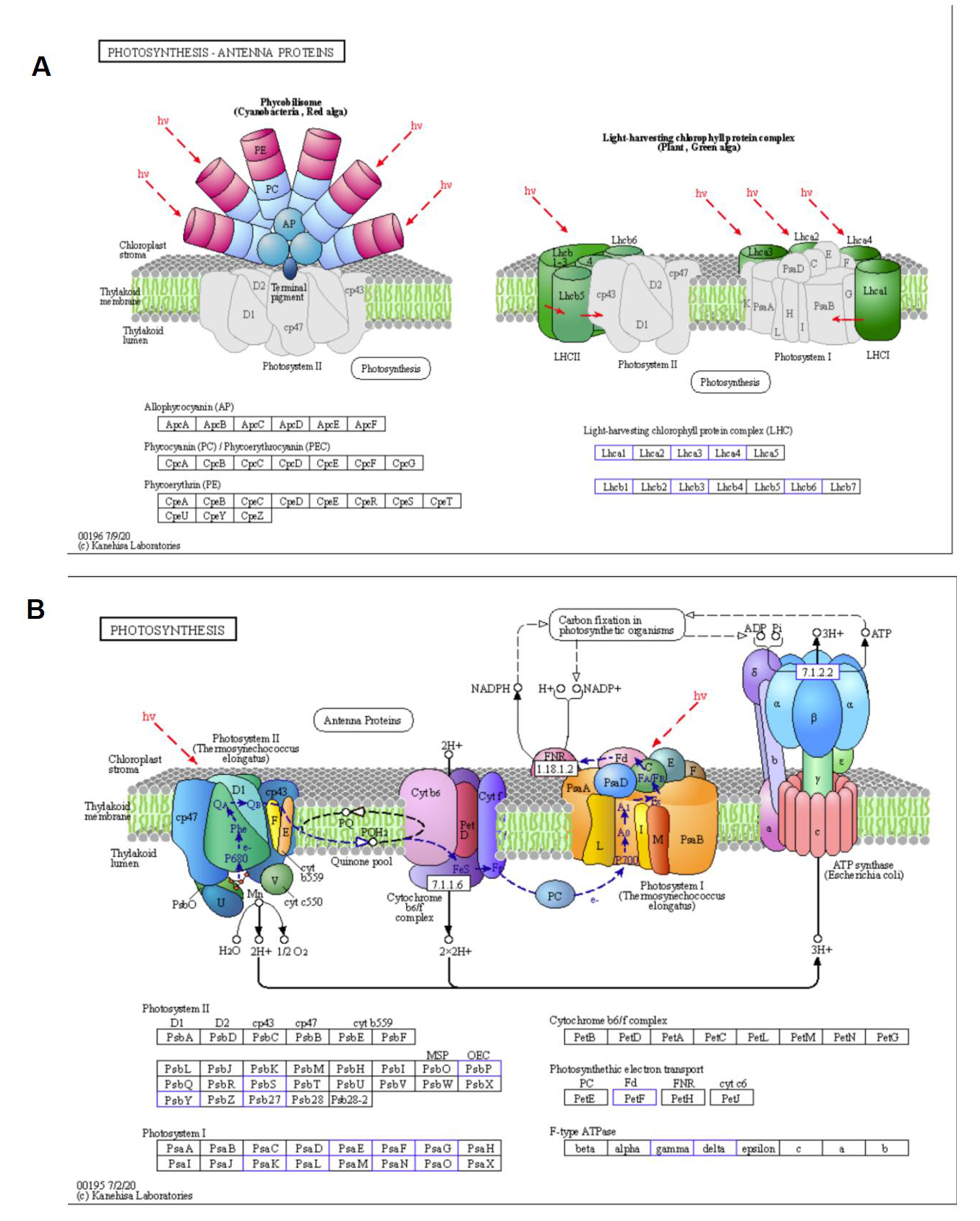

Supplement: Supplementary file 2 — Supplementary Material 2 [file 12870_2025_7180_MOESM2_ESM.jpg]

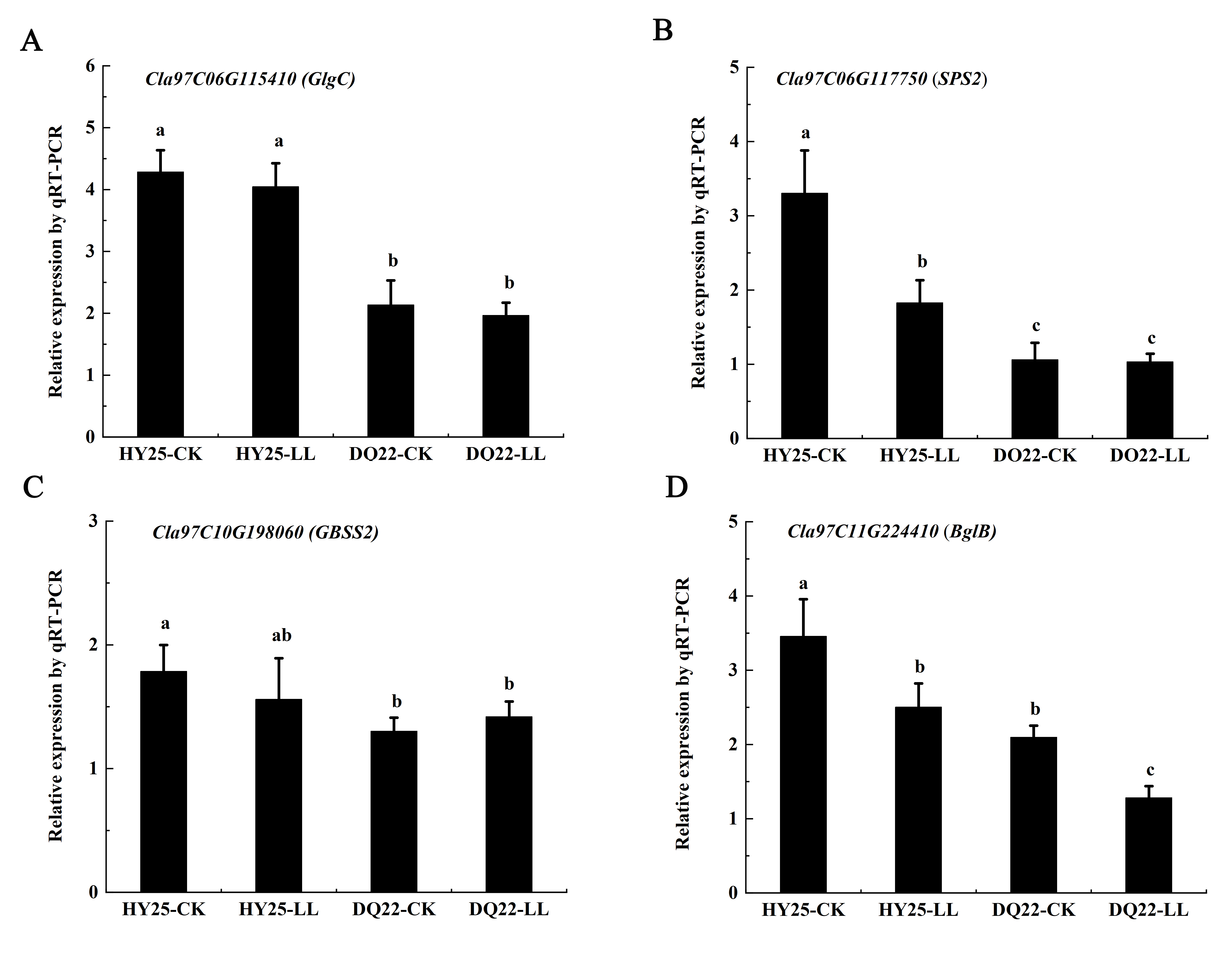

Supplement: Supplementary file 3 — Supplementary Material 3 [file 12870_2025_7180_MOESM3_ESM.jpg]

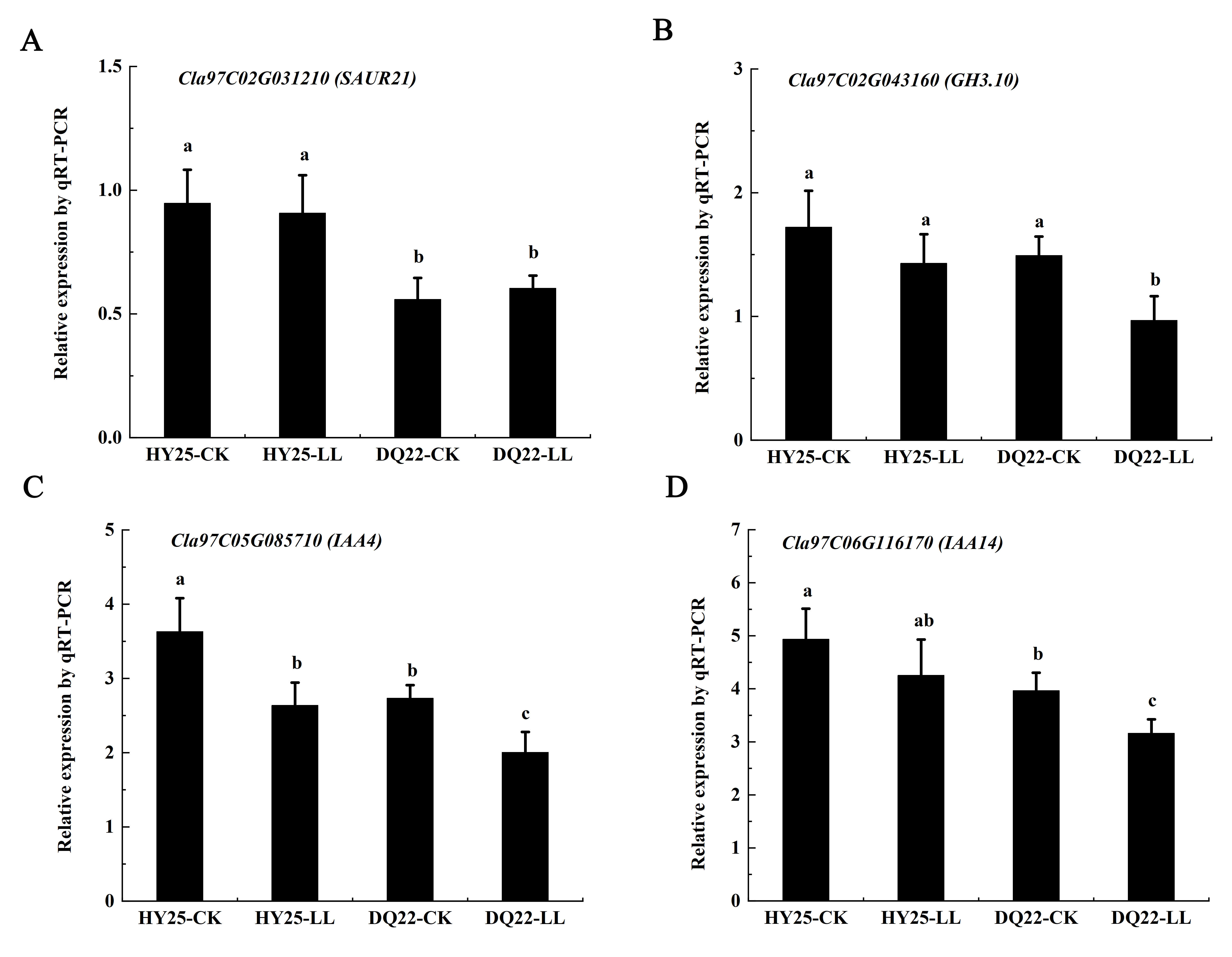

Supplement: Supplementary file 4 — Supplementary Material 4 [file 12870_2025_7180_MOESM4_ESM.jpg]
